# Supplementary material for: The Small RNA Universe of Capitella teleta
Source: Front Mol Biosci. 2022 Feb 25;9:802814. doi: 10.3389/fmolb.2022.802814 (PMC8915122; doi:10.3389/fmolb.2022.802814)
Supplement: Supplementary file 1 [file DataSheet1.ZIP › Supplement/known/CAPTEscaffold_548_17647.pdf]

| | | |
|---|---|---|
| Provisional ID | : | CAPTEscaffold_548_17647 |
| Score total | : | 1157472.2 |
| Score for star read(s) | : | 3.9 |
| Score for read counts | : | 1157461.7 |
| Score for mfe | : | 1.9 |
| Score for randfold | : | 1.6 |
| Score for cons. seed | : | 3 |
| Total read count | : | 2270323 |
| Mature read count | : | 2078722 |
| Loop read count | : | 0 |
| Star read count | : | 191601 |

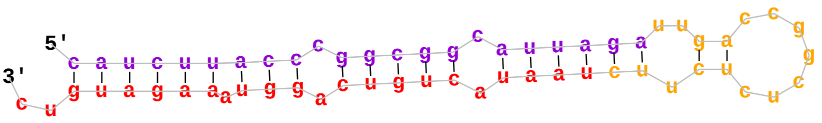


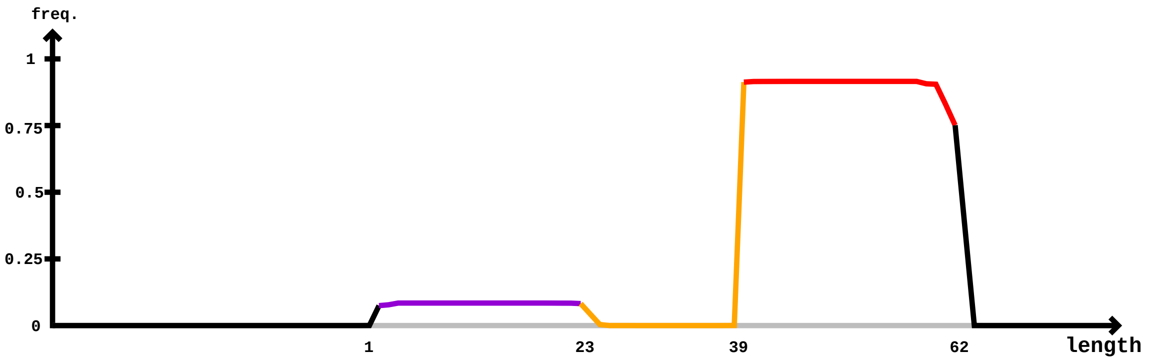


```
                                      Star                                    Mature
5'- cgcugagagcagccagccgcggguuguguggacaucuuacccggcggcauuagauugaccggcucucuucuaauacugucagguaaagaugucuccacgacuccauguc  -3'  obs
    cgcugagagcagccagccgcggguuguguggacaucuuacccggcggcauuagauugaccggcucucuucuaauacugucagguaaagaugucuccacgacuccauguc       exp
    cgcugagagcagccagccgcggguuguguggacaucuuacccggcggcauuagauugaccggcucucuucuaauacugucagguaaagaugucuccacgacuccauguc       known
    .((((........))))...((((((((.((((((((((((.(((((.((((((..((.......)).)))))).))))).))).))))))))).))))))))......   reads   mm   sample

    ............ccagccgcggguugugugga.............................................................................   1       0    seq
    .............cagccgcggguugugugga.............................................................................   1       0    seq
    ...............................acaucuuacccggcggcauu..........................................................   4       0    seq
    ...............................acaucuuGcccggcggcauu..........................................................   1       1    seq
    ...............................acaucuuacccggcggcauua.........................................................   12      0    seq
    ...............................acaucuuGcccggcggcauua.........................................................   8       1    seq
    ...............................Gcaucuuacccggcggcauuag........................................................   1       1    seq
    ...............................acaucuuacccggcggcauuGg........................................................   1       1    seq
    ...............................acaucuuGcccggcggcauuag........................................................   5       1    seq
    ...............................Ccaucuuacccggcggcauuag........................................................   3       1    seq
    ...............................acaucuuacccggcggcauuag........................................................   331     0    seq
    ...............................acauAuuacccggcggcauuag........................................................   1       1    seq
    ...............................acaucuuacccggcggcauuaga.......................................................   101     0    seq
    ...............................acaucuuacccggcggcauuagU.......................................................   18      1    seq
    ...............................Ccaucuuacccggcggcauuaga.......................................................   4       1    seq
    ................................caucuuacccggcggcGu...........................................................   1       1    seq
    ................................caucuAacccggcggcau...........................................................   1       1    seq
    ................................caucuuaccAggcggcau...........................................................   2       1    seq
    ................................caucuCacccggcggcau...........................................................   1       1    seq
    ................................caucuuacccggcggcau...........................................................   231     0    seq
    ................................caucuuaccUggcggcau...........................................................   1       1    seq
    ................................caucuuacccggcggcauA..........................................................   1       1    seq
    ................................caucuuacAcggcggcauu..........................................................   1       1    seq
    ................................caucuuacccAgcggcauu..........................................................   2       1    seq
    ................................Aaucuuacccggcggcauu..........................................................   1       1    seq
    ................................caucuuacccggcggcauu..........................................................   434     0    seq
    ................................caucuuacccggcggcaAu..........................................................   1       1    seq
    ................................Uaucuuacccggcggcauu..........................................................   1       1    seq
    ................................caucuuacAcggcggcauua.........................................................   1       1    seq
    ................................caucuuGcccggcggcauua.........................................................   72      1    seq
    ................................Uaucuuacccggcggcauua.........................................................   1       1    seq
```

Star Mature

```
cgcugagagcagccagccgcggguuguguggacaucuuacccggcggcauuagauugaccggcucucuucuaauacugucagguaaagaugucuccacgacuccauguc
................................caAcuuacccggcggcauua.........................................................   1     1   seq
................................caucuuacccggcggcauua.........................................................   167   0   seq
................................caucuCacccggcggcauua.........................................................   2     1   seq
................................caucuuCcccggcggcauuag........................................................   2     1   seq
................................caucuuacccggcUgcauuag........................................................   2     1   seq
................................caucuuacccUgcggcauuag........................................................   1     1   seq
................................caucuuacccggcgAcauuag........................................................   1     1   seq
................................caucuuacccggcggcUuuag........................................................   1     1   seq
................................caucuuacccggcggcauuaA........................................................   9     1   seq
................................cGucuuacccggcggcauuag........................................................   1     1   seq
................................caucuuaccAggcggcauuag........................................................   3     1   seq
................................caucAuacccggcggcauuag........................................................   3     1   seq
................................Uaucuuacccggcggcauuag........................................................   2     1   seq
................................caucuuacccggcggcauCag........................................................   1     1   seq
................................caucuuacccggcgUcauuag........................................................   2     1   seq
................................caucuuacccggcAgcauuag........................................................   1     1   seq
................................caucuuacccggcggcaGuag........................................................   1     1   seq
................................caucuuacccAgcggcauuag........................................................   3     1   seq
................................caucuuacccgCcggcauuag........................................................   1     1   seq
................................caucuuacccggcggcauuaU........................................................   7     1   seq
................................caucuuacccggcggcauuag........................................................   2822  0   seq
................................caucuuaUccggcggcauuag........................................................   1     1   seq
................................caucuuacccggcggcauuGg........................................................   1     1   seq
................................caucuuacccggAggcauuag........................................................   2     1   seq
................................caucuuacccggcggcauuUg........................................................   1     1   seq
................................caucuuacAcggcggcauuag........................................................   1     1   seq
................................caucuuacccggcggUauuag........................................................   1     1   seq
................................Aaucuuacccggcggcauuag........................................................   1     1   seq
................................caucuuaccUggcggcauuag........................................................   2     1   seq
................................caucuuacccgCcggcauuaga.......................................................   12    1   seq
................................caucuuacccggcggcauuGga.......................................................   35    1   seq
................................caucuuacccggcUgcauuaga.......................................................   15    1   seq
................................caucuuaccGggcggcauuaga.......................................................   14    1   seq
................................caucuuacccggcAgcauuaga.......................................................   77    1   seq
................................cauUuuacccggcggcauuaga.......................................................   10    1   seq
................................caAcuuacccggcggcauuaga.......................................................   168   1   seq
................................caucuuacccggcgAcauuaga.......................................................   21    1   seq
................................caucuuaccAggcggcauuaga.......................................................   123   1   seq
................................Gaucuuacccggcggcauuaga.......................................................   9     1   seq
................................caGcuuacccggcggcauuaga.......................................................   2     1   seq
................................caucuuacccggcggcauuaCa.......................................................   7     1   seq
................................caucuuCcccggcggcauuaga.......................................................   6     1   seq
................................caucuuacccggcggcauuagC.......................................................   4     1   seq
................................caucuuacccggAggcauuaga.......................................................   68    1   seq
................................caucuAacccggcggcauuaga.......................................................   59    1   seq
................................Aaucuuacccggcggcauuaga.......................................................   61    1   seq
................................caucuuacccggcggcauuagU.......................................................   448   1   seq
................................caucuuUcccggcggcauuaga.......................................................   3     1   seq
................................caucuuacccggcggcaGuaga.......................................................   17    1   seq
................................Uaucuuacccggcggcauuaga.......................................................   264   1   seq
................................caucuuacccggcggcaAuaga.......................................................   21    1   seq
................................caucuuacccggcggcaCuaga.......................................................   12    1   seq
................................caucuuacccggcggGauuaga.......................................................   17    1   seq
................................caucGuacccggcggcauuaga.......................................................   5     1   seq
................................caucuuacccggcggcauuUga.......................................................   21    1   seq
................................caucuuacccggcggcauuCga.......................................................   1     1   seq
................................Naucuuacccggcggcauuaga.......................................................   40    1   seq
................................caucuuaccUggcggcauuaga.......................................................   131   1   seq
................................caucuCacccggcggcauuaga.......................................................   17    1   seq
................................caucuuacUcggcggcauuaga.......................................................   47    1   seq
................................caucuuacccgAcggcauuaga.......................................................   17    1   seq
................................caucuuacccggGggcauuaga.......................................................   7     1   seq
................................caucuuacccggcggcauuagG.......................................................   124   1   seq
................................caucuuacccggcggcauuaNa.......................................................   3     1   seq
................................caucuuacccgUcggcauuaga.......................................................   8     1   seq
................................caucuuNcccggcggcauuaga.......................................................   1     1   seq
................................caucuuacccggcCgcauuaga.......................................................   6     1   seq
................................caucuuacccggcggNauuaga.......................................................   1     1   seq
................................caucuuacGcggcggcauuaga.......................................................   12    1   seq
................................caCcuuacccggcggcauuaga.......................................................   115   1   seq
```

**Star** **Mature**

```
cgcugagagcagccagccgcggguuguguggacaucuuacccggcggcauuagauugaccggcucucuucuaauacugucagguaaagaugucuccacgacuccauguc
................................caucuuacccggcggcauuaAa.......................................................  21      1  seq
................................caucuuaGccggcggcauuaga.......................................................  2       1  seq
................................cauGuuacccggcggcauuaga.......................................................  3       1  seq
................................caucuuacccggcggcGuuaga.......................................................  93      1  seq
................................cNucuuacccggcggcauuaga.......................................................  3       1  seq
................................caucuuacccggUggcauuaga.......................................................  40      1  seq
................................caucuuacccggcggAauuaga.......................................................  32      1  seq
................................caucuuacAcggcggcauuaga.......................................................  173     1  seq
................................caucuuacccggcggcauuaga.......................................................  161811  0  seq
................................caucuuacccggcgCcauuaga.......................................................  6       1  seq
................................caucAuacccggcggcauuaga.......................................................  119     1  seq
................................caucuuacccggcgUcauuaga.......................................................  15      1  seq
................................caucuuacccAgcggcauuaga.......................................................  132     1  seq
................................cauAuuacccggcggcauuaga.......................................................  18      1  seq
................................caucuuacccggcggcUuuaga.......................................................  24      1  seq
................................caucuuacccUgcggcauuaga.......................................................  22      1  seq
................................caucuuaUccggcggcauuaga.......................................................  10      1  seq
................................caucuuacccggcggcauAaga.......................................................  23      1  seq
................................caucuNacccggcggcauuaga.......................................................  4       1  seq
................................caucCuacccggcggcauuaga.......................................................  13      1  seq
................................caucuuacccggcggUauuaga.......................................................  23      1  seq
................................cGucuuacccggcggcauuaga.......................................................  26      1  seq
................................caucuuaAccggcggcauuaga.......................................................  38      1  seq
................................caucuuGcccggcggcauuaga.......................................................  327     1  seq
................................cUucuuacccggcggcauuaga.......................................................  9       1  seq
................................caucuuacccCgcggcauuaga.......................................................  7       1  seq
................................caucuuacccggcggcauGaga.......................................................  12      1  seq
................................caucuuacccggcggcauCaga.......................................................  17      1  seq
................................caucuuacccggcggcauuaUa.......................................................  14      1  seq
................................caucuuacccggcggcauuagaA......................................................  264     1  seq
................................caucuuacccggcggcauuagau......................................................  7       0  seq
................................caucuuacccggcggcauuagauu.....................................................  7       0  seq
................................caucuuacccggcggcauuagaAu.....................................................  4       1  seq
.................................aucuuacccggcggcauu..........................................................  16      0  seq
.................................aucuuacccggcggcauua.........................................................  15      0  seq
.................................Gucuuacccggcggcauuag........................................................  4       1  seq
.................................aucuuacccggcggcauuaU........................................................  3       1  seq
.................................aucuuaAccggcggcauuag........................................................  2       1  seq
.................................aucuuacccggcggcauuag........................................................  311     0  seq
.................................aucuuacccggcgCcauuag........................................................  1       1  seq
.................................aucuuacccAgcggcauuag........................................................  2       1  seq
.................................aucuuacccggcUgcauuaga.......................................................  1       1  seq
.................................aucuuacccggcggcGuuaga.......................................................  4       1  seq
.................................aucuuacccAgcggcauuaga.......................................................  8       1  seq
.................................Gucuuacccggcggcauuaga.......................................................  121     1  seq
.................................aucuuacccggcggcauCaga.......................................................  1       1  seq
.................................aucuuacccggcggAauuaga.......................................................  2       1  seq
.................................aucAuacccggcggcauuaga.......................................................  2       1  seq
.................................aucuCacccggcggcauuaga.......................................................  1       1  seq
.................................aucuuacccggcggcaAuaga.......................................................  2       1  seq
.................................aucuuacccCgcggcauuaga.......................................................  2       1  seq
.................................aucuuacccUgcggcauuaga.......................................................  1       1  seq
.................................aucuAacccggcggcauuaga.......................................................  5       1  seq
.................................auAuuacccggcggcauuaga.......................................................  6       1  seq
.................................aucuuaUccggcggcauuaga.......................................................  2       1  seq
.................................auUuuacccggcggcauuaga.......................................................  2       1  seq
.................................aucuuacccggcgAcauuaga.......................................................  1       1  seq
.................................aucuuacccggcggcauAaga.......................................................  1       1  seq
.................................aucuuacUcggcggcauuaga.......................................................  3       1  seq
.................................aucuuacccggcggcauuaga.......................................................  6631    0  seq
.................................aucuuacccggcggcauuagG.......................................................  3       1  seq
.................................aucuuacAcggcggcauuaga.......................................................  7       1  seq
.................................aucuuacccggAggcauuaga.......................................................  5       1  seq
.................................aucuuacccggcAgcauuaga.......................................................  2       1  seq
.................................aucuuGcccggcggcauuaga.......................................................  43      1  seq
.................................aucuuacccggUggcauuaga.......................................................  1       1  seq
.................................aucuuacccggcggcauuagU.......................................................  3       1  seq
.................................aucuuaccAggcggcauuaga.......................................................  1       1  seq
.................................aAcuuacccggcggcauuaga.......................................................  4       1  seq
.................................aucuuaAccggcggcauuaga.......................................................  5       1  seq
```

```
                                    Star                                   Mature
cgcugagagcagccagccgcgguuguguuggacaucuuacccggcggcauuagauugaccggcucucuucuaauacugucagguaaagaugucuccacgacuccauguc
.................................Uucuuacccggcggcauuaga.......................................................	1	1	seq
.................................aucuuaccUggcggcauuaga.......................................................	8	1	seq
.................................aucuuacccggcggcauuaUa.......................................................	1	1	seq
.................................aucuuGcccggcggcauuagau......................................................	2	1	seq
.................................Gucuuacccggcggcauuagau......................................................	2	1	seq
.................................auUuuacccggcggcauuagau......................................................	1	1	seq
.................................aucuuacccggcggcauuagau......................................................	25	0	seq
.................................aucuuacccggcggcauuagaA......................................................	4	1	seq
.................................Cucuuacccggcggcauuagauu.....................................................	2	1	seq
.................................aucuuacccggcggcauuagauu.....................................................	2	0	seq
..................................ucuuacccggcggcauuaC........................................................	1	1	seq
..................................ucuuacAcggcggcauuag........................................................	3	1	seq
..................................Acuuacccggcggcauuag........................................................	1	1	seq
..................................ucuuacccggcggcauuag........................................................	139	0	seq
..................................ucuuaccUggcggcauuaga.......................................................	2	1	seq
..................................ucuuacccggcggcauCaga.......................................................	2	1	seq
..................................ucuuacccggcggcauuagG.......................................................	6	1	seq
..................................ucuuacccggcggUauuaga.......................................................	1	1	seq
..................................Ncuuacccggcggcauuaga.......................................................	5	1	seq
..................................ucuuaccAggcggcauuaga.......................................................	3	1	seq
..................................ucuAacccggcggcauuaga.......................................................	1	1	seq
..................................ucuuaGccggcggcauuaga.......................................................	1	1	seq
..................................ucuuacccggcggcaAuaga.......................................................	1	1	seq
..................................ucuuacccggcAgcauuaga.......................................................	4	1	seq
..................................ucuuacccggcggcGuuaga.......................................................	1	1	seq
..................................ucuuacccggcggcauuagU.......................................................	13	1	seq
..................................ucuuGcccggcggcauuaga.......................................................	3	1	seq
..................................ucuuacccAgcggcauuaga.......................................................	5	1	seq
..................................Gcuuacccggcggcauuaga.......................................................	3	1	seq
..................................ucuuaAccggcggcauuaga.......................................................	1	1	seq
..................................ucuuacccggcggcauuGga.......................................................	1	1	seq
..................................ucCuacccggcggcauuaga.......................................................	1	1	seq
..................................ucAuacccggcggcauuaga.......................................................	8	1	seq
..................................ucuuacAcggcggcauuaga.......................................................	15	1	seq
..................................ucuuacccggcggcaGuaga.......................................................	1	1	seq
..................................ucuuacccUgcggcauuaga.......................................................	1	1	seq
..................................ucuuacUcggcggcauuaga.......................................................	1	1	seq
..................................ucuuacccggcUgcauuaga.......................................................	1	1	seq
..................................Acuuacccggcggcauuaga.......................................................	11	1	seq
..................................ucuuacccggcggcauuaga.......................................................	4629	0	seq
..................................ucuuacccggcggcauuagau......................................................	869	0	seq
..................................ucuuacccggcggUauuagau......................................................	1	1	seq
..................................ucGuacccggcggcauuagau......................................................	1	1	seq
..................................ucuuacccggcggcauuagGu......................................................	1	1	seq
..................................ucuuacccggcggcaAuagau......................................................	2	1	seq
..................................ucuGacccggcggcauuagau......................................................	1	1	seq
..................................Acuuacccggcggcauuagau......................................................	3	1	seq
..................................ucuuacccUgcggcauuagau......................................................	1	1	seq
..................................ucuuaUccggcggcauuagau......................................................	1	1	seq
..................................ucuuacAcggcggcauuagau......................................................	5	1	seq
..................................ucuuacccCgcggcauuagau......................................................	1	1	seq
..................................ucuuaccUggcggcauuagau......................................................	1	1	seq
..................................ucuuacccggUggcauuagau......................................................	1	1	seq
..................................ucuuacccggcggcauuagaA......................................................	15	1	seq
..................................ucuuacccAgcggcauuagau......................................................	2	1	seq
..................................ucuuacccggAggcauuagauu.....................................................	3	1	seq
..................................ucuuacccggcggcauuaAauu.....................................................	2	1	seq
..................................ucuuacccAgcggcauuagauu.....................................................	15	1	seq
..................................ucuuacccggcggcauuagaAu.....................................................	4	1	seq
..................................Ncuuacccggcggcauuagauu.....................................................	5	1	seq
..................................Ccuuacccggcggcauuagauu.....................................................	2	1	seq
..................................ucuuacccggcggcauAagauu.....................................................	1	1	seq
..................................ucuuacAcggcggcauuagauu.....................................................	29	1	seq
..................................ucuuacccgUcggcauuagauu.....................................................	1	1	seq
..................................ucuuaccUggcggcauuagauu.....................................................	11	1	seq
..................................ucuuacccggGggcauuagauu.....................................................	1	1	seq
..................................ucuuacccggcggcauuagauu.....................................................	8368	0	seq
..................................ucuuacccggcggcaAuagauu.....................................................	2	1	seq
..................................ucuuacccggcAgcauuagauu.....................................................	7	1	seq
..................................ucuuacccggcggGauuagauu.....................................................	2	1	seq
```

**Star** **Mature**

```
cgcugagagcagccagccgcggguuguguggacaucuuacccggcggcauuagauugaccggcucucuucuaauacugucagguaaagaugucuccacgacuccauguc
..................................uGuuacccggcggcauuagauu.....................................................   1    1   seq
..................................ucCuacccggcggcauuagauu.....................................................   1    1   seq
..................................ucuuacccggcggcGuuagauu.....................................................   1    1   seq
..................................ucuuacccggcggcauuGgauu.....................................................   1    1   seq
..................................ucuuaUccggcggcauuagauu.....................................................   1    1   seq
..................................ucuAacccggcggcauuagauu.....................................................   2    1   seq
..................................ucuuacccggUggcauuagauu.....................................................   3    1   seq
..................................ucuuacccggcggcauuagaCu.....................................................   1    1   seq
..................................ucuuaAccggcggcauuagauu.....................................................   2    1   seq
..................................ucuuacccggcggcauuagaGu.....................................................   1    1   seq
..................................ucuuGcccggcggcauuagauu.....................................................   10   1   seq
..................................uAuuacccggcggcauuagauu.....................................................   1    1   seq
..................................ucuuacccggcggcaGuagauu.....................................................   1    1   seq
..................................ucuuacccggcggcauuUgauu.....................................................   1    1   seq
..................................ucuuacccgAcggcauuagauu.....................................................   2    1   seq
..................................ucuuacccggcgUcauuagauu.....................................................   1    1   seq
..................................ucuuacccggcgAcauuagauu.....................................................   2    1   seq
..................................Gcuuacccggcggcauuagauu.....................................................   4    1   seq
..................................ucuuacccCgcggcauuagauu.....................................................   1    1   seq
..................................ucuuacccggcggcauuagauG.....................................................   5    1   seq
..................................ucuuacccggcggcauuagauC.....................................................   2    1   seq
..................................ucuuaccAggcggcauuagauu.....................................................   3    1   seq
..................................ucuuacUcggcggcauuagauu.....................................................   5    1   seq
..................................ucAuacccggcggcauuagauu.....................................................   7    1   seq
..................................ucuuacccggcggcauGagauu.....................................................   1    1   seq
..................................Acuuacccggcggcauuagauu.....................................................   28   1   seq
..................................ucuCacccggcggcauuagauu.....................................................   3    1   seq
..................................ucuuacccggcggcauuagauA.....................................................   24   1   seq
..................................ucuuacccggcggcauuagauug....................................................   55   0   seq
..................................ucuuacccggcggcauuagauuU....................................................   3    1   seq
..................................ucuuacccggcggcauuagauuA....................................................   9    1   seq
..................................ucuuacccggcggcauuagauuC....................................................   1    1   seq
..................................Acuuacccggcggcauuagauuga...................................................   1    1   seq
..................................ucuuacccggcggcauuagauuAa...................................................   4    1   seq
..................................ucuuacccggcggcauuagauuga...................................................   104  0   seq
..................................ucuuacccggcggcauuagGuuga...................................................   1    1   seq
..................................ucuuacccggcggcauuagauugac..................................................   5    0   seq
..................................ucuuacccggcggcauuagauugaA..................................................   88   1   seq
..................................ucuuacccggcggcauuagauugacc.................................................   49   0   seq
..................................ucuuacccggcggcauuagaAugacc.................................................   1    1   seq
...................................cuuacccggcggcauuaga.......................................................   1    0   seq
....................................uuacccggcggcauuaga.......................................................   61   0   seq
....................................uuacccggcggcauuagauu.....................................................   25   0   seq
...................................................................Aucuaauacugucagguaaagauguc................   2    1   seq
...................................................................Aucuaauacugucagguaaagaugucu...............   3    1   seq
....................................................................ucuaauGcugucagguaaag.....................   1    1   seq
....................................................................ucuaauGcugucagguaaaga....................   41   1   seq
....................................................................ucuaauacugucagguaaaga....................   14   0   seq
....................................................................ucuaauacugucGgguaaaga....................   3    1   seq
....................................................................ucuaauacugucagguaaagau...................   4    0   seq
....................................................................ucuaauGcugucagguaaagau...................   49   1   seq
....................................................................uGuaauacugucagguaaagau...................   1    1   seq
....................................................................ucuaauacugucagguaaagaug..................   23   0   seq
....................................................................ucuaauGcugucagguaaagaug..................   41   1   seq
....................................................................ucuaauGcugucagguaaagaugu.................   1    1   seq
....................................................................ucuaauacugucagguaaagaugu.................   1    0   seq
....................................................................ucuaauacugucagguaaagauguc................   14   0   seq
....................................................................ucuaauacugucGgguaaagauguc................   3    1   seq
.....................................................................cuaauacugucagguaaaga....................   1    0   seq
.....................................................................cuaauGcugucagguaaaga....................   5    1   seq
.....................................................................cuaauGcugucagguaaagau...................   21   1   seq
.....................................................................cuaauGcugucagguaaagaug..................   45   1   seq
.....................................................................Uuaauacugucagguaaagaug..................   4    1   seq
.....................................................................cuaauacugucagguaaagaug..................   19   0   seq
.....................................................................cAaauacugucagguaaagaugu.................   1    1   seq
.....................................................................cuaauacugucagguaaagaugu.................   29   0   seq
.....................................................................cuaauGcugucagguaaagaugu.................   73   1   seq
.....................................................................Nuaauacugucagguaaagaugu.................   1    1   seq
.....................................................................cuaauacugucGgguaaagaugu.................   1    1   seq
.....................................................................Uuaauacugucagguaaagaugu.................   1    1   seq
```

```
                              Star                                  Mature
cgcugagagcagccagccgcggguugugugga caucuuacccggcggcauuaga uugaccggcucucuuc uaauacugucagguaaagauguc uccacgacuccauguc
.....................................................................Auaauacugucagguaaagauguc................   8      1   seq
.....................................................................cuaauacugucagguaaagauguc................   317    0   seq
.....................................................................cuaauacugucagguaaagauguA................   1      1   seq
.....................................................................Uuaauacugucagguaaagauguc................   1      1   seq
.....................................................................cuaauGcugucagguaaagauguc................   30     1   seq
.....................................................................cuaauacugucagguaaagaugucu...............   5      0   seq
.....................................................................cuaauGcugucagguaaagaugucu...............   4      1   seq
.....................................................................Auaauacugucagguaaagaugucu...............   1      1   seq
......................................................................uaauGcugucagguaaag.....................   81     1   seq
......................................................................uaauacugucagguaaaA.....................   9      1   seq
......................................................................uaauacugucagguaaag.....................   172    0   seq
......................................................................Aaauacugucagguaaag.....................   1      1   seq
......................................................................uaauacugucagguaaaU.....................   2      1   seq
......................................................................uaauacuguGagguaaaga....................   2      1   seq
......................................................................uaauacuguUagguaaaga....................   3      1   seq
......................................................................Caauacugucagguaaaga....................   3      1   seq
......................................................................uaauacuguAagguaaaga....................   12     1   seq
......................................................................uaauacugAcagguaaaga....................   5      1   seq
......................................................................uaauCcugucagguaaaga....................   1      1   seq
......................................................................uaauacuCucagguaaaga....................   3      1   seq
......................................................................uaGuacugucagguaaaga....................   1      1   seq
......................................................................uaauacugucagguaaGga....................   2      1   seq
......................................................................uaauaGugucagguaaaga....................   1      1   seq
......................................................................Aaauacugucagguaaaga....................   74     1   seq
......................................................................uGauacugucagguaaaga....................   5      1   seq
......................................................................uaauacugucagguaaUga....................   1      1   seq
......................................................................uaaAacugucagguaaaga....................   7      1   seq
......................................................................uaauacugCcagguaaaga....................   5      1   seq
......................................................................uaauacugucagguGaaga....................   2      1   seq
......................................................................uaauacugucagguaaagC....................   1      1   seq
......................................................................uaauacuAucagguaaaga....................   2      1   seq
......................................................................Naauacugucagguaaaga....................   2      1   seq
......................................................................uaauacugucagguaaagG....................   11     1   seq
......................................................................uaauacugucagUuaaaga....................   3      1   seq
......................................................................uaauacugucagguaaaUa....................   1      1   seq
......................................................................uaauacugucUgguaaaga....................   1      1   seq
......................................................................uaauaAugucagguaaaga....................   4      1   seq
......................................................................uaauacugucagguaaagU....................   5      1   seq
......................................................................uaauacugucagguaaaga....................   12627  0   seq
......................................................................uaauacugucaAguaaaga....................   15     1   seq
......................................................................uaauacugGcagguaaaga....................   1      1   seq
......................................................................uaauacCgucagguaaaga....................   1      1   seq
......................................................................uaauacugucaCguaaaga....................   1      1   seq
......................................................................uaauGcugucagguaaaga....................   6987   1   seq
......................................................................uaauacugucaggGaaaga....................   1      1   seq
......................................................................uaauacugucagguaGaga....................   5      1   seq
......................................................................uaauacugucagAuaaaga....................   2      1   seq
......................................................................uaauacAgucagguaaaga....................   13     1   seq
......................................................................Gaauacugucagguaaaga....................   2      1   seq
......................................................................uaauacugucagCuaaaga....................   2      1   seq
......................................................................uaauacugucaggAaaaga....................   2      1   seq
......................................................................uaauacugucGgguaaaga....................   201    1   seq
......................................................................uaauacugAcagguaaagau...................   1      1   seq
......................................................................uaauacuguGagguaaagau...................   1      1   seq
......................................................................Aaauacugucagguaaagau...................   18     1   seq
......................................................................uaauacAgucagguaaagau...................   3      1   seq
......................................................................uaauaAugucagguaaagau...................   2      1   seq
......................................................................uaauGcugucagguaaagau...................   1128   1   seq
......................................................................uaaAacugucagguaaagau...................   2      1   seq
......................................................................uaauacugucagguaaagaA...................   31     1   seq
......................................................................uaauacugucaggCaaagau...................   2      1   seq
......................................................................uaauacuguUagguaaagau...................   1      1   seq
......................................................................uaauacugucagAuaaagau...................   1      1   seq
......................................................................uaauacugucaAguaaagau...................   1      1   seq
......................................................................Gaauacugucagguaaagau...................   1      1   seq
......................................................................uaauacugucUgguaaagau...................   1      1   seq
......................................................................uaauacuCucagguaaagau...................   2      1   seq
......................................................................Naauacugucagguaaagau...................   1      1   seq
......................................................................uaauacugucagguaaagaC...................   1      1   seq
......................................................................uaauacuguAagguaaagau...................   1      1   seq
```

**Star** **Mature**

| | | | |
|---|---|---|---|
| cgcugagagcagccagccgcggguuguguggacaucuuacccggcggcauuagauugaccggcucucuucuaauacugucagguaaagaugucuccacgacuccauguc | | | |
| ......................................................................uaUuacugucagguaaagau................... | 1 | 1 | seq |
| ......................................................................uaauacugucagguaaagau................... | 2629 | 0 | seq |
| ......................................................................uaauacugucGgguaaagau................... | 46 | 1 | seq |
| ......................................................................uNauacugucagguaaagaug.................. | 4 | 1 | seq |
| ......................................................................uaauacugucagguaaaUaug.................. | 5 | 1 | seq |
| ......................................................................uaauacuUucagguaaagaug.................. | 14 | 1 | seq |
| ......................................................................uaauacugucagguaaagGug.................. | 12 | 1 | seq |
| ......................................................................uaauaNugucagguaaagaug.................. | 2 | 1 | seq |
| ......................................................................uaaGacugucagguaaagaug.................. | 6 | 1 | seq |
| ......................................................................uGauacugucagguaaagaug.................. | 57 | 1 | seq |
| ......................................................................uaauacugucagguCaagaug.................. | 1 | 1 | seq |
| ......................................................................uaauacugucagguaaagauC.................. | 32 | 1 | seq |
| ......................................................................uaauacuCucagguaaagaug.................. | 26 | 1 | seq |
| ......................................................................uUauacugucagguaaagaug.................. | 2 | 1 | seq |
| ......................................................................uaauacugAcagguaaagaug.................. | 51 | 1 | seq |
| ......................................................................uaauacugucagguaaagaCg.................. | 16 | 1 | seq |
| ......................................................................uaauacuguNagguaaagaug.................. | 1 | 1 | seq |
| ......................................................................uaauacugucagguGaagaug.................. | 29 | 1 | seq |
| ......................................................................uaauacugucagguaaagauA.................. | 376 | 1 | seq |
| ......................................................................uaauacuguGagguaaagaug.................. | 9 | 1 | seq |
| ......................................................................uaauacugucagguaaGgaug.................. | 67 | 1 | seq |
| ......................................................................uCauacugucagguaaagaug.................. | 2 | 1 | seq |
| ......................................................................uaauacugucagAuaaagaug.................. | 17 | 1 | seq |
| ......................................................................uaauacugucaUguaaagaug.................. | 7 | 1 | seq |
| ......................................................................uaauacugucGgguaaagaug.................. | 796 | 1 | seq |
| ......................................................................uaauaGugucagguaaagaug.................. | 4 | 1 | seq |
| ......................................................................uaauacugucagguaaagCug.................. | 2 | 1 | seq |
| ......................................................................uaauacugucagCuaaagaug.................. | 9 | 1 | seq |
| ......................................................................uaauacugucagguaGagaug.................. | 23 | 1 | seq |
| ......................................................................uaGuacugucagguaaagaug.................. | 14 | 1 | seq |
| ......................................................................uaauacugucagguaaagaAg.................. | 26 | 1 | seq |
| ......................................................................uaauacugCcagguaaagaug.................. | 16 | 1 | seq |
| ......................................................................uaauacugucaggCaaagaug.................. | 21 | 1 | seq |
| ......................................................................uaauaAugucagguaaagaug.................. | 87 | 1 | seq |
| ......................................................................uaauacugucaggGaaagaug.................. | 4 | 1 | seq |
| ......................................................................uaauacugucaggAaaagaug.................. | 29 | 1 | seq |
| ......................................................................uaauGcugucagguaaagaug.................. | 35569 | 1 | seq |
| ......................................................................uaauCcugucagguaaagaug.................. | 2 | 1 | seq |
| ......................................................................Gaauacugucagguaaagaug.................. | 58 | 1 | seq |
| ......................................................................uaauacugucagguaaaAaug.................. | 4 | 1 | seq |
| ......................................................................uaauacugucagguaaUgaug.................. | 14 | 1 | seq |
| ......................................................................uaauacugGcagguaaagaug.................. | 8 | 1 | seq |
| ......................................................................uaauaUugucagguaaagaug.................. | 5 | 1 | seq |
| ......................................................................uaauacugucagguaaNgaug.................. | 1 | 1 | seq |
| ......................................................................uaauacugucagguaaaCaug.................. | 3 | 1 | seq |
| ......................................................................uaNuacugucagguaaagaug.................. | 1 | 1 | seq |
| ......................................................................uaauacugucagguaaagauN.................. | 1 | 1 | seq |
| ......................................................................uaauacugucagguUaagaug.................. | 2 | 1 | seq |
| ......................................................................uaauacAgucagguaaagaug.................. | 79 | 1 | seq |
| ......................................................................uaauUcugucagguaaagaug.................. | 11 | 1 | seq |
| ......................................................................uaauacugucagguaUagaug.................. | 9 | 1 | seq |
| ......................................................................uaauacuguAagguaaagaug.................. | 94 | 1 | seq |
| ......................................................................uaauacuAucagguaaagaug.................. | 17 | 1 | seq |
| ......................................................................uaauacuguUagguaaagaug.................. | 36 | 1 | seq |
| ......................................................................Caauacugucagguaaagaug.................. | 31 | 1 | seq |
| ......................................................................uaaAacugucagguaaagaug.................. | 140 | 1 | seq |
| ......................................................................uaauacugucUgguaaagaug.................. | 13 | 1 | seq |
| ......................................................................uaauacugucaAguaaagaug.................. | 49 | 1 | seq |
| ......................................................................uaauacugucagguaaagUug.................. | 9 | 1 | seq |
| ......................................................................uaauacugucagguaCagaug.................. | 3 | 1 | seq |
| ......................................................................uaaCacugucagguaaagaug.................. | 11 | 1 | seq |
| ......................................................................uaauacCgucagguaaagaug.................. | 19 | 1 | seq |
| ......................................................................Naauacugucagguaaagaug.................. | 38 | 1 | seq |
| ......................................................................uaauacugucaCguaaagaug.................. | 4 | 1 | seq |
| ......................................................................uaauacugucagguaaagaGg.................. | 26 | 1 | seq |
| ......................................................................uaauacugucagguaaagaug.................. | 131117 | 0 | seq |
| ......................................................................Aaauacugucagguaaagaug.................. | 665 | 1 | seq |
| ......................................................................uaauacugucagguaaagauU.................. | 18 | 1 | seq |
| ......................................................................uaUuacugucagguaaagaug.................. | 13 | 1 | seq |
| ......................................................................uaauacugucCgguaaagaug.................. | 3 | 1 | seq |

```
                                Star                                Mature
cgcugagagcagccagccgcggguuguguggacaucuuacccggcggcauuagauugaccggcucucuucuaauacugucagguaaagaugucuccacgacuccauguc
......................................................................uaauacGgucagguaaagaug..................  15      1   seq
......................................................................uaauacugucagUuaaagaug..................  6       1   seq
......................................................................uaauUcugucagguaaagaugu.................  15      1   seq
......................................................................uaUuacugucagguaaagaugu.................  8       1   seq
......................................................................uaauaGugucagguaaagaugu.................  10      1   seq
......................................................................uaauacugucaggCaaagaugu.................  15      1   seq
......................................................................uaauacugucagguaaagCugu.................  4       1   seq
......................................................................uaauacugucaggAaaagaugu.................  36      1   seq
......................................................................uaaGacugucagguaaagaugu.................  9       1   seq
......................................................................Gaauacugucagguaaagaugu.................  72      1   seq
......................................................................uaauacuAucagguaaagaugu.................  25      1   seq
......................................................................uaauacugucagguaaagaugA.................  363     1   seq
......................................................................uaauacAgucagguaaagaugu.................  119     1   seq
......................................................................uaauacugucagguaaagaugu.................  146286  0   seq
......................................................................uaauacugucagguaaaUaugu.................  15      1   seq
......................................................................uaauacugucagguaGagaugu.................  37      1   seq
......................................................................uaauacugucGgguaaagaugu.................  1668    1   seq
......................................................................uaauacugGcagguaaagaugu.................  13      1   seq
......................................................................uaauaUugucagguaaagaugu.................  3       1   seq
......................................................................uaGuacugucagguaaagaugu.................  14      1   seq
......................................................................uaauacugucagCuaaagaugu.................  13      1   seq
......................................................................uaauacuguUagguaaagaugu.................  55      1   seq
......................................................................uaauacugucagguaaagauNu.................  1       1   seq
......................................................................uaauacugucagguaaagauCu.................  20      1   seq
......................................................................uaauacugucagguaaagaugG.................  145     1   seq
......................................................................uaauacGgucagguaaagaugu.................  22      1   seq
......................................................................uaauacugucagguaaagGugu.................  29      1   seq
......................................................................uaauacugAcagguaaagaugu.................  47      1   seq
......................................................................uaauacugucagguaaagaugC.................  21      1   seq
......................................................................uaauaNugucagguaaagaugu.................  1       1   seq
......................................................................uaaAacugucagguaaagaugu.................  142     1   seq
......................................................................uaauacugucagUuaaagaugu.................  8       1   seq
......................................................................uaauacugucagguaaagUugu.................  10      1   seq
......................................................................Aaauacugucagguaaagaugu.................  624     1   seq
......................................................................uaauacugucagguaaagaCgu.................  14      1   seq
......................................................................uaauacugucagguUaagaugu.................  7       1   seq
......................................................................uaaCacugucagguaaagaugu.................  30      1   seq
......................................................................uUauacugucagguaaagaugu.................  2       1   seq
......................................................................uaauacCgucagguaaagaugu.................  22      1   seq
......................................................................Naauacugucagguaaagaugu.................  44      1   seq
......................................................................uaNuacugucagguaaagaugu.................  2       1   seq
......................................................................uaauaAugucagguaaagaugu.................  63      1   seq
......................................................................uaauacugucagguaaUgaugu.................  20      1   seq
......................................................................uaauacugucagguaaGgaugu.................  141     1   seq
......................................................................uaauacuguGagguaaagaugu.................  10      1   seq
......................................................................uaauacuUucagguaaagaugu.................  18      1   seq
......................................................................uaauacugucagAuaaagaugu.................  18      1   seq
......................................................................uaauacugucaCguaaagaugu.................  7       1   seq
......................................................................uaauacugucagguaaagaAgu.................  28      1   seq
......................................................................uaauacuCucagguaaagaugu.................  11      1   seq
......................................................................uaauacuguAagguaaagaugu.................  117     1   seq
......................................................................uaauacugucagguaaagaGgu.................  9       1   seq
......................................................................uaauacugucagguaaagauUu.................  11      1   seq
......................................................................uaauacugucUgguaaagaugu.................  11      1   seq
......................................................................uaauacugCcagguaaagaugu.................  14      1   seq
......................................................................uaauacugucaggGaaagaugu.................  6       1   seq
......................................................................uaauacugucaAguaaagaugu.................  48      1   seq
......................................................................uaauacugucagguaUagaugu.................  11      1   seq
......................................................................uaauGcugucagguaaagaugu.................  28150   1   seq
......................................................................uaauacugucagguaaaCaugu.................  5       1   seq
......................................................................uGauacugucagguaaagaugu.................  88      1   seq
......................................................................Caauacugucagguaaagaugu.................  28      1   seq
......................................................................uaauacugucaUguaaagaugu.................  22      1   seq
......................................................................uaauacugucCgguaaagaugu.................  2       1   seq
......................................................................uaauacugucagguaaagauAu.................  20      1   seq
......................................................................uaauacugucagguaCagaugu.................  1       1   seq
......................................................................uNauacugucagguaaagaugu.................  3       1   seq
......................................................................uaauCcugucagguaaagaugu.................  2       1   seq
......................................................................uaauacugucagguaaaAaugu.................  16      1   seq
......................................................................uaauacugucagguGaagaugu.................  33      1   seq
```

**Star** **Mature**

| | | | |
|---|---|---|---|
| cgcugagagcagccagccgcggguuguguggacaucuuacccggcggcauuagauugaccggcucucuucuaauacugucagguaaagaugucuccacgacuccauguc | | | |
| ......................................................................uaaGacugucagguaaagauguc................ | 55 | 1 | seq |
| ......................................................................uaauacugucagAuaaagauguc................ | 166 | 1 | seq |
| ......................................................................uaauacugucGgguaaagauguc................ | 7299 | 1 | seq |
| ......................................................................uaauacugucagguCaagauguc................ | 6 | 1 | seq |
| ......................................................................uaGuacugucagguaaagauguc................ | 190 | 1 | seq |
| ......................................................................uaaAacugucagguaaagauguc................ | 1227 | 1 | seq |
| ......................................................................uaauacugucCgguaaagauguc................ | 33 | 1 | seq |
| ......................................................................uaauacugucaAguaaagauguc................ | 865 | 1 | seq |
| ......................................................................uaauGcugucagguaaagauguc................ | 168724 | 1 | seq |
| ......................................................................uaauacugucagguaaagauguc................ | 1402636 | 0 | seq |
| ......................................................................uaauacugucagguaGagauguc................ | 184 | 1 | seq |
| ......................................................................uaauacugCcagguaaagauguc................ | 196 | 1 | seq |
| ......................................................................uaauacAgucagguaaagauguc................ | 1009 | 1 | seq |
| ......................................................................Naauacugucagguaaagauguc................ | 418 | 1 | seq |
| ......................................................................uaauacugucagguaaGgauguc................ | 1135 | 1 | seq |
| ......................................................................uaauacugucagguaaagaugGc................ | 44 | 1 | seq |
| ......................................................................uaauacugucagguaaagGuguc................ | 170 | 1 | seq |
| ......................................................................uaauacNgucagguaaagauguc................ | 1 | 1 | seq |
| ......................................................................uaauaNugucagguaaagauguc................ | 12 | 1 | seq |
| ......................................................................uaauacugucagguaaagCuguc................ | 19 | 1 | seq |
| ......................................................................uaauacugucagguaaagaugAc................ | 634 | 1 | seq |
| ......................................................................uUauacugucagguaaagauguc................ | 38 | 1 | seq |
| ......................................................................uaauacugucagguaaagaugNc................ | 1 | 1 | seq |
| ......................................................................uaauacugucagguaaagaAguc................ | 389 | 1 | seq |
| ......................................................................uaauacugucagguaaagauguA................ | 3050 | 1 | seq |
| ......................................................................uaauacuUucagguaaagauguc................ | 97 | 1 | seq |
| ......................................................................uaauCcugucagguaaagauguc................ | 22 | 1 | seq |
| ......................................................................uaauacugucagguaaagauNuc................ | 3 | 1 | seq |
| ......................................................................uaauaGugucagguaaagauguc................ | 55 | 1 | seq |
| ......................................................................uaauacugucagCuaaagauguc................ | 150 | 1 | seq |
| ......................................................................uaauacugucagguaaagauguN................ | 7 | 1 | seq |
| ......................................................................uaauaAugucagguaaagauguc................ | 429 | 1 | seq |
| ......................................................................uaUuacugucagguaaagauguc................ | 107 | 1 | seq |
| ......................................................................uGauacugucagguaaagauguc................ | 620 | 1 | seq |
| ......................................................................uaauacugucagguaaagaGguc................ | 59 | 1 | seq |
| ......................................................................Caauacugucagguaaagauguc................ | 287 | 1 | seq |
| ......................................................................uaauacGgucagguaaagauguc................ | 172 | 1 | seq |
| ......................................................................uaauacugucagguaCagauguc................ | 8 | 1 | seq |
| ......................................................................uCauacugucagguaaagauguc................ | 9 | 1 | seq |
| ......................................................................uaauacuAucagguaaagauguc................ | 234 | 1 | seq |
| ......................................................................uaauaUugucagguaaagauguc................ | 97 | 1 | seq |
| ......................................................................uNauacugucagguaaagauguc................ | 59 | 1 | seq |
| ......................................................................uaauacugucagUuaaagauguc................ | 112 | 1 | seq |
| ......................................................................uaauacugucagguaaaUauguc................ | 94 | 1 | seq |
| ......................................................................Aaauacugucagguaaagauguc................ | 6085 | 1 | seq |
| ......................................................................uaCuacugucagguaaagauguc................ | 6 | 1 | seq |
| ......................................................................uaauUcugucagguaaagauguc................ | 153 | 1 | seq |
| ......................................................................uaauacugucagguaaagauCuc................ | 112 | 1 | seq |
| ......................................................................uaauacugGcagguaaagauguc................ | 111 | 1 | seq |
| ......................................................................uaaCacugucagguaaagauguc................ | 133 | 1 | seq |
| ......................................................................uaauacugucagguaaagaCguc................ | 179 | 1 | seq |
| ......................................................................uaauacugucUgguaaagauguc................ | 154 | 1 | seq |
| ......................................................................uaauacuguUagguaaagauguc................ | 464 | 1 | seq |
| ......................................................................uaauacugNcagguaaagauguc................ | 2 | 1 | seq |
| ......................................................................uaauacugucaCguaaagauguc................ | 47 | 1 | seq |
| ......................................................................uaauacugucagguaaagauguU................ | 871 | 1 | seq |
| ......................................................................uaauacugucaggCaaagauguc................ | 202 | 1 | seq |
| ......................................................................uaauacugAcagguaaagauguc................ | 530 | 1 | seq |
| ......................................................................uaauacugucaggGaaagauguc................ | 62 | 1 | seq |
| ......................................................................uaauacuguGagguaaagauguc................ | 102 | 1 | seq |
| ......................................................................uaauacuCucagguaaagauguc................ | 217 | 1 | seq |
| ......................................................................uaauacCgucagguaaagauguc................ | 216 | 1 | seq |
| ......................................................................uaauacugucaggNaaagauguc................ | 1 | 1 | seq |
| ......................................................................uaauacugucagguaaagauUuc................ | 85 | 1 | seq |
| ......................................................................uaNuacugucagguaaagauguc................ | 5 | 1 | seq |
| ......................................................................uaauacugucaUguaaagauguc................ | 128 | 1 | seq |
| ......................................................................uaauacugucagguaaaAauguc................ | 148 | 1 | seq |
| ......................................................................uaauacugucagguaaagauguG................ | 69 | 1 | seq |
| ......................................................................uaauacugucagguaaaCauguc................ | 127 | 1 | seq |
| ......................................................................Gaauacugucagguaaagauguc................ | 661 | 1 | seq |

```
                                Star                                   Mature
cgcugagagcagccagccgcggguugugugga caucuuacccggcggcauuaga uugaccggcucucuuc uaauacugucagguaaagauguc uccacgacuccauguc
......................................................................uaauacugucNgguaaagauguc................    1       1    seq
......................................................................uaauacugucagguaaagaugCc................    159     1    seq
......................................................................uaauacuguNagguaaagauguc................    3       1    seq
......................................................................uaauacugucagguGaagauguc................    347     1    seq
......................................................................uaauacugucagguaUagauguc................    113     1    seq
......................................................................uaauacugucagguaaCgauguc................    6       1    seq
......................................................................uaauacugucagguaaagauAuc................    168     1    seq
......................................................................uaauacugucagguaaUgauguc................    164     1    seq
......................................................................uaauacugucaggAaaagauguc................    320     1    seq
......................................................................uaauacugucagguaaagUuguc................    87      1    seq
......................................................................uaauacugucagguaaNgauguc................    1       1    seq
......................................................................uaauacuguAagguaaagauguc................    1298    1    seq
......................................................................uaauacugucagguUaagauguc................    66      1    seq
......................................................................uCauacugucagguaaagaugucu...............    1       1    seq
......................................................................uaauacugucaCguaaagaugucu...............    2       1    seq
......................................................................uaauacugucagCuaaagaugucu...............    5       1    seq
......................................................................uaauacugucaggCaaagaugucu...............    7       1    seq
......................................................................Gaauacugucagguaaagaugucu...............    22      1    seq
......................................................................uaauacugucagguaaagUugucu...............    5       1    seq
......................................................................uaauacugucUgguaaagaugucu...............    6       1    seq
......................................................................uaauacugucagguaaagauguGu...............    4       1    seq
......................................................................uaauacugucagguaaagGugucu...............    11      1    seq
......................................................................uaGuacugucagguaaagaugucu...............    6       1    seq
......................................................................uaauacugucagguaaagaugucG...............    91      1    seq
......................................................................uaauacuUucagguaaagaugucu...............    4       1    seq
......................................................................uNauacugucagguaaagaugucu...............    4       1    seq
......................................................................uaauGcugucagguaaagaugucu...............    13533   1    seq
......................................................................uaauacugucagguaaagauUucu...............    3       1    seq
......................................................................Aaauacugucagguaaagaugucu...............    322     1    seq
......................................................................uaauacugucaggAaaagaugucu...............    19      1    seq
......................................................................uaauacugucGgguaaagaugucu...............    1494    1    seq
......................................................................uaauacugAcagguaaagaugucu...............    27      1    seq
......................................................................uaauacugucagAuaaagaugucu...............    11      1    seq
......................................................................uaauacugucagguaaagaugucA...............    6240    1    seq
......................................................................uaauacugucCgguaaagaugucu...............    3       1    seq
......................................................................uaUuacugucagguaaagaugucu...............    2       1    seq
......................................................................uaauacugucagguaaUgaugucu...............    5       1    seq
......................................................................uaauacugucaggGaaagaugucu...............    1       1    seq
......................................................................uaauacugucagguaaagauAucu...............    8       1    seq
......................................................................uaauacugucagguaaagaugAcu...............    29      1    seq
......................................................................uaauacugucagguaaGgaugucu...............    233     1    seq
......................................................................uaauacuguAagguaaagaugucu...............    46      1    seq
......................................................................uaauacugucagguGaagaugucu...............    18      1    seq
......................................................................uaauacugucagguaGagaugucu...............    8       1    seq
......................................................................uUauacugucagguaaagaugucu...............    2       1    seq
......................................................................uaauacugGcagguaaagaugucu...............    5       1    seq
......................................................................uaauacugucagguaaagaCgucu...............    5       1    seq
......................................................................uaauacugucagguaaagauguUu...............    31      1    seq
......................................................................uaauacuguGagguaaagaugucu...............    6       1    seq
......................................................................uaauacugucagguaaaCaugucu...............    3       1    seq
......................................................................uaauacugucagguaaagaugCcu...............    2       1    seq
......................................................................uaauacuCucagguaaagaugucu...............    11      1    seq
......................................................................uaauUcugucagguaaagaugucu...............    7       1    seq
......................................................................uaaAacugucagguaaagaugucu...............    58      1    seq
......................................................................uaauacAgucagguaaagaugucu...............    47      1    seq
......................................................................uaauaGugucagguaaagaugucu...............    1       1    seq
......................................................................uaauacugucagguaaagaugucu...............    70962   0    seq
......................................................................uaauacCgucagguaaagaugucu...............    18      1    seq
......................................................................uaauacuAucagguaaagaugucu...............    10      1    seq
......................................................................uaauacugucagUuaaagaugucu...............    3       1    seq
......................................................................uaauacugucagguaaagaugucC...............    58      1    seq
......................................................................uaaCacugucagguaaagaugucu...............    7       1    seq
......................................................................Naauacugucagguaaagaugucu...............    22      1    seq
......................................................................uaauacugucaAguaaagaugucu...............    23      1    seq
......................................................................uaauacugucagguaUagaugucu...............    6       1    seq
......................................................................uaauacugucagguaaagaugGcu...............    4       1    seq
......................................................................uaauacugucagguaaaAaugucu...............    8       1    seq
......................................................................uaauacugucagguUaagaugucu...............    2       1    seq
......................................................................uaauacugucagguaaagaGgucu...............    12      1    seq
......................................................................uaauaAugucagguaaagaugucu...............    27      1    seq
```

Star Mature

| | | | |
|---|---|---|---|
| cgcugagagcagccagccgcggguuguggacaucuuacccggcggcauuagauugaccggcucucuucuaauacugucagguaaagaugucuccacgacuccauguc | | | |
| ....................................................................uaauacugCcagguaaagaugucu............... | 12 | 1 | seq |
| ....................................................................uGauacugucagguaaagaugucu............... | 39 | 1 | seq |
| ....................................................................uaaGacugucagguaaagaugucu............... | 6 | 1 | seq |
| ....................................................................uaauacugucagguaaagauguAu............... | 73 | 1 | seq |
| ....................................................................uaauacugucagguaaagaugucN............... | 2 | 1 | seq |
| ....................................................................Caauacugucagguaaagaugucu............... | 9 | 1 | seq |
| ....................................................................uaauacuguUagguaaagaugucu............... | 31 | 1 | seq |
| ....................................................................uaauacugucagguaaagauCucu............... | 5 | 1 | seq |
| ....................................................................uaauacugucagguaaaUaugucu............... | 10 | 1 | seq |
| ....................................................................uaauacGgucagguaaagaugucu............... | 12 | 1 | seq |
| ....................................................................uaauacugucagguaaagaAgucu............... | 15 | 1 | seq |
| ....................................................................uaauaUugucagguaaagaugucu............... | 2 | 1 | seq |
| ....................................................................uaauacugucaUguaaagaugucu............... | 3 | 1 | seq |
| ....................................................................uaauaNugucagguaaagaugucu............... | 2 | 1 | seq |
| ....................................................................uaauacugucagguaaCgaugucu............... | 1 | 1 | seq |
| ....................................................................uaauacugucagguaaagaugucuU.............. | 208 | 1 | seq |
| ....................................................................uaauacugucagguaaagaugucAc.............. | 3 | 1 | seq |
| ....................................................................uaauacugucagguaaagaugucuc.............. | 1 | 0 | seq |
| ....................................................................uaauacugucagguaaagaugucuA.............. | 1 | 1 | seq |
| .....................................................................aauacugucagguaaaga.................... | 4 | 0 | seq |
| .....................................................................aauGcugucagguaaaga.................... | 1 | 1 | seq |
| .....................................................................Uauacugucagguaaaga.................... | 1 | 1 | seq |
| .....................................................................aauGcugucagguaaagau................... | 4 | 1 | seq |
| .....................................................................Uauacugucagguaaagaug.................. | 17 | 1 | seq |
| .....................................................................aauacugucagguaaagaug.................. | 24 | 0 | seq |
| .....................................................................aauGcugucagguaaagaug.................. | 15 | 1 | seq |
| .....................................................................aauacugucagguaaagaugA................. | 1 | 1 | seq |
| .....................................................................aauacugucagguaaagaugu................. | 28 | 0 | seq |
| .....................................................................Uauacugucagguaaagaugu................. | 16 | 1 | seq |
| .....................................................................aauGcugucagguaaagaugu................. | 20 | 1 | seq |
| .....................................................................aauacuguUagguaaagauguc................ | 1 | 1 | seq |
| .....................................................................aauGcugucagguaaagauguc................ | 58 | 1 | seq |
| .....................................................................Gauacugucagguaaagauguc................ | 1 | 1 | seq |
| .....................................................................aauacAgucagguaaagauguc................ | 1 | 1 | seq |
| .....................................................................aauacugucagguaaagauguc................ | 330 | 0 | seq |
| .....................................................................Uauacugucagguaaagauguc................ | 127 | 1 | seq |
| .....................................................................aauacugucagguaaagauguU................ | 1 | 1 | seq |
| .....................................................................aauacugucagguaaagaugucA............... | 10 | 1 | seq |
| .....................................................................aauacugucagguaaUgaugucu............... | 1 | 1 | seq |
| .....................................................................aauacugucagguaaagUugucu............... | 1 | 1 | seq |
| .....................................................................aauacugucagguaaGgaugucu............... | 4 | 1 | seq |
| .....................................................................aauacuguUagguaaagaugucu............... | 1 | 1 | seq |
| .....................................................................Uauacugucagguaaagaugucu............... | 8 | 1 | seq |
| .....................................................................aauacAgucagguaaagaugucu............... | 4 | 1 | seq |
| .....................................................................aauacugucaggAaaagaugucu............... | 2 | 1 | seq |
| .....................................................................aauGcugucagguaaagaugucu............... | 1007 | 1 | seq |
| .....................................................................aauacugucagguaaagaugAcu............... | 3 | 1 | seq |
| .....................................................................aauacugucUgguaaagaugucu............... | 1 | 1 | seq |
| .....................................................................aauacugucagUuaaagaugucu............... | 1 | 1 | seq |
| .....................................................................Nauacugucagguaaagaugucu............... | 2 | 1 | seq |
| .....................................................................aauacugucagguaaagaugucG............... | 3 | 1 | seq |
| .....................................................................aauacugucagguaaagaugucC............... | 4 | 1 | seq |
| .....................................................................aauacugucGgguaaagaugucu............... | 65 | 1 | seq |
| .....................................................................aauacugucagguaGagaugucu............... | 3 | 1 | seq |
| .....................................................................aauacugucagguaaagaugucu............... | 3576 | 0 | seq |
| .....................................................................aauacugucaAguaaagaugucu............... | 1 | 1 | seq |
| .....................................................................aCuacugucagguaaagaugucu............... | 1 | 1 | seq |
| .....................................................................aauacugCcagguaaagaugucu............... | 1 | 1 | seq |
| .....................................................................aauacugucagguaaagaCgucu............... | 1 | 1 | seq |
| .....................................................................aaAacugucagguaaagaugucu............... | 11 | 1 | seq |
| .....................................................................aauacugucagguaaagaAgucu............... | 1 | 1 | seq |
| .....................................................................aauacugAcagguaaagaugucu............... | 3 | 1 | seq |
| .....................................................................aauacugucagguaaagauguGu............... | 1 | 1 | seq |
| .....................................................................aauacuAucagguaaagaugucu............... | 1 | 1 | seq |
| .....................................................................aauacugucagguaaagaugucuU.............. | 2 | 1 | seq |
| ......................................................................auGcugucagguaaagaug.................. | 1 | 1 | seq |
| ......................................................................auacugucagguaaagaug.................. | 6 | 0 | seq |
| ......................................................................auacugucagguaaagaugu................. | 10 | 0 | seq |
| ......................................................................auGcugucagguaaagaugu................. | 1 | 1 | seq |
| ......................................................................auGcugucagguaaagauguc................ | 12 | 1 | seq |

Star Mature

```
cgcugagagcagccagccgcggguugugugga caucuuacccggcggcauuaga uugaccggcucucuuc uaauacugucagguaaagauguc uccacgacuccauguc
........................................................................Uuacugucagguaaagauguc................	2	1	seq
........................................................................auacugucagguaaagauguc................	74	0	seq
........................................................................auacugAcagguaaagaugucu...............	1	1	seq
........................................................................auacugucagguaaagaugucu...............	37	0	seq
........................................................................auacugucGgguaaagaugucu...............	1	1	seq
........................................................................auGcugucagguaaagaugucu...............	276	1	seq
.........................................................................uacugucagguaaagaug..................	5	0	seq
.........................................................................uacugucagguaaagaugu.................	4	0	seq
.........................................................................uGcugucagguaaagaugu.................	1	1	seq
.........................................................................uacugucagguaaagauguc................	159	0	seq
.........................................................................uGcugucagguaaagauguc................	12	1	seq
.........................................................................uacugucagguaUagaugucu...............	1	1	seq
.........................................................................uacugucagguaaagaugucu...............	1	0	seq
.........................................................................uacugucagguaaagaugucucc.............	6	0	seq
..........................................................................acugucagguaaagaugu.................	32	0	seq
..........................................................................Gcugucagguaaagaugu.................	22	1	seq
..........................................................................acugucagguaaagauAu.................	1	1	seq
..........................................................................acugucagguaaagauguc................	135	0	seq
..........................................................................Gcugucagguaaagauguc................	16	1	seq
..........................................................................acugucagguaaagCuguc................	1	1	seq
...........................................................................cugucagguaaagauguc................	197	0	seq
...........................................................................cAgucagguaaagauguc................	1	1	seq
...........................................................................cugucagguaaagaCguc................	1	1	seq
...........................................................................cugucagguaaagaugucu...............	1	0	seq
............................................................................ugucagguaaagaugucu...............	1	0	seq
```
